# Differences in gut microbiota between Dutch and South-Asian Surinamese: potential implications for type 2 diabetes mellitus

**DOI:** 10.1038/s41598-024-54769-4

**Published:** 2024-02-26

**Authors:** Eric I. Nayman, Brooke A. Schwartz, Michaela Polmann, Alayna C. Gumabong, Max Nieuwdorp, Trevor Cickovski, Kalai Mathee

**Affiliations:** 1 Department of Human and Molecular Genetics, Herbert Wertheim College of Medicine, Florida International University, Miami, FL USA; 2 Bioinformatics Research Group, Knight Foundation School of Computing and Information Sciences, College of Engineering and Computing, Florida International University, Miami, FL USA; 3 Amsterdam Diabetes Center, Department of Internal Medicine, Academic Medical Center, VU University Medical Center, Amsterdam, The Netherlands; 4 Biomolecular Sciences Institute, Florida International University, Miami, FL USA

**Keywords:** Microbiome, Metagenomics, Microbial ecology, Biomarkers, Ecological networks, Diabetes

## Abstract

Gut microbiota, or the collection of diverse microorganisms in a specific ecological niche, are known to significantly impact human health. Decreased gut microbiota production of short-chain fatty acids (SCFAs) has been implicated in type 2 diabetes mellitus (T2DM) disease progression. Most microbiome studies focus on ethnic majorities. This study aims to understand how the microbiome differs between an ethnic majority (the Dutch) and minority (the South-Asian Surinamese (SAS)) group with a lower and higher prevalence of T2DM, respectively. Microbiome data from the Healthy Life in an Urban Setting (HELIUS) cohort were used. Two age- and gender-matched groups were compared: the Dutch (n = 41) and SAS (n = 43). Microbial community compositions were generated via DADA2. Metrics of microbial diversity and similarity between groups were computed. Biomarker analyses were performed to determine discriminating taxa. Bacterial co-occurrence networks were constructed to examine ecological patterns. A tight microbiota cluster was observed in the Dutch women, which overlapped with some of the SAS microbiota. The Dutch gut contained a more interconnected microbial ecology, whereas the SAS network was dispersed, i.e., contained fewer inter-taxonomic correlational relationships. *Bacteroides caccae, Butyricicoccus, Alistipes putredinis, Coprococcus comes*, *Odoribacter splanchnicus,* and *Lachnospira* were enriched in the Dutch gut. *Haemophilus*, *Bifidobacterium,* and *Anaerostipes hadrus* discriminated the SAS gut. All but *Lachnospira* and certain strains of *Haemophilus* are known to produce SCFAs. The Dutch gut microbiome was distinguished from the SAS by diverse, differentially abundant SCFA-producing taxa with significant cooperation. The dynamic ecology observed in the Dutch was not detected in the SAS. Among several potential gut microbial biomarkers, *Haemophilus parainfluenzae* likely best characterizes the ethnic minority group, which is more predisposed to T2DM. The higher prevalence of T2DM in the SAS may be associated with the gut dysbiosis observed.

## Introduction

A microbiome is a community of phylogenetically diverse microorganisms and their multi-omic content that inhabit a specific ecological niche, such as the human gut^1^. The microbiome and the host are embedded in a mutually dependent relationship that impacts host behavior and microbial community structure and function^2^. As host-microbe interactions shape reciprocal fitness, phenotype, and metabolism, the host and the microbiome coevolve^2,3^. *Dysbiosis*, or pathologic alteration of the baseline microbial milieu, may drive and/or result from disease progression^4,5^. Our resident microbial flora has been widely recognized as a key but not yet fully understood mediator in the pathophysiology of many communicable and noncommunicable diseases^5–11^, including type 2 diabetes mellitus (T2DM)^12–16^.

Diabetes is a chronic, metabolic disease in which hyperglycemia leads to multi-organ damage over time^17^. Since the 1990s, the global prevalence of T2DM has increased, and the age-adjusted prevalence is expected to rise from 6.3% in 2019 to 7.8% in 2045 across Europe^18^. Disproportionately high rates of diabetes and related complications affect migrant and ethnic minority groups living in Western societies^19^. Diet, genetic predisposition, body weight, and sedentary lifestyle are key factors in the multifaceted pathophysiology of T2DM^20^. Recently, the gut microbiome of diabetics has been shown to be distinctly different from that of normoglycemic, insulin-sensitive individuals^14–16,21^. Most agree that the abundances of *Ruminococcus, Fusobacterium*, and *Blautia* are positively correlated with T2DM while *Bifidobacterium, Bacteroides, Faecalibacterium, Akkermansia,* and *Roseburia* are negatively correlated with T2DM^13,22–24^. However, a large knowledge gap still exists: how do the quantitative presences of the many other gut inhabitants vary with hyperglycemia and insulin resistance? For example, many disagree on the nature of the correlative relationship between T2DM and the abundance of *Lactobacillus*^23^.

Short-chain fatty acids (SCFAs), namely, acetate, butyrate, and propionate, and the microbes that produce them have been of particular interest in the diabetic gut because of the favorable effect that these molecules have on host function^25–27^. Most of the beneficial effects of SCFAs on glucose metabolism and insulin signaling are mediated via the GPR41 and GPR43 receptors^27–29^. These molecules can activate intestinal gluconeogenesis^27,30^, potentiate glucose-stimulated insulin secretion through glucagon like peptide-1 (GLP-1) dependent and independent pathways^28,29,31^, and attenuate the chronic release of pro-inflammatory cytokines that worsens insulin resistance^32–34^. SCFAs have many other important functions on host metabolism, which are well described by recent reviews^26,27^. Ultimately, a decrease in SCFA-producing taxa is thought to be comorbid with T2DM and may cause or worsen the disease. Of note, cross-feeding between SCFA-producing taxa plays a major role in the gut microbial functional ecology. For example, *Bacteroides thetaiotaomicron, Blautia obeum, Roseburia inulinivorans, Listeria* sp., and *Clostridium sphenoides* can all create a major intermediate metabolite (1,2-propanediol) which can be used by *Lactobacillus reuteri* to make propionate. *Roseburia* can then take up acetate, the most widely produced SCFA, and make butyrate from glucose^35^.

Ethnicity and place of habitation are thought to play an even larger role than metabolic health in shaping gut microbiome composition^36–38^. Ethnicity is a particularly important factor as it connotes similar diet, shared genetics, and migration patterns, all of which have their own variable impact on gut microbial flora. The multi-ethnic Healthy Life in an Urban Setting (HELIUS) prospective cohort study estimated that the impact of ethnicity on gut microbiome composition is ~ 6%^37^. The HELIUS cohort included participants from the six major ethnic groups living in Amsterdam, the Netherlands at the time of sample collection. Of these ethnicities, the Dutch, Ghanaian, and South-Asian Surinamese (SAS) were found to have the most discriminant gut microbiomes^37^. In the Dutch population, the ethnic majority of Amsterdam, nine core gut bacterial species were identified: *Subdoligranulum* sp., *Alistipes onderdonkii*, *Alistipes putredinis*, *Alistipes shahii*, *Bacteroides uniformis*, *Bacteroides vulgatus*, *Eubacterium rectale*, *Faecalibacterium prausnitzii* and *Oscillibacter* sp. These were also consistently found across many other populations^38^. Of these, several were shown to be significantly depleted, e.g., *Faecalibacterium prausnitzii,* and enriched, e.g., *Bacteroides* sp., in the SAS as compared to the Dutch^37^.

The aim of our study is to compare the gut microbiome ecology between the Dutch and SAS groups, as these had the lowest and highest prevalence of T2DM among the HELIUS ethnicities, respectively. Our objective is to characterize and replicate, as microbiome studies are notoriously challenged by reproducibility, the bacterial biomarkers and inter-taxa relationships between these two groups. Network analysis was performed to compare the gut microbiomes of the two ethnicities because it can elucidate co-occurrence patterns, which can identify relationships between different bacterial groups. We demonstrate differences in the SCFA-producing taxa and correlate these with primarily ethnicity and secondarily metabolic health.

## Methods

### Genomic data source

Our dataset is from a multi-ethnic prospective cohort study, the HELIUS study. This cohort is composed of five major ethnic groups (Surinamese, Dutch, Ghanaian, Moroccan**,** and Turkish) aged 18–70 years old. Participants were randomly invited, and then stratified by ethnicity. All were living in Amsterdam, the Netherlands at the time of sample collection (2011–2015). A total of 2170 stool samples, each from a different individual, were collected, and the metagenomes were sent for sequencing of the 16S rRNA V4 hypervariable region. This was done on a 2 × 250 base-pair (bp) MiSeq system with use of the 515F and 806R primers. The 2170 sequenced fecal samples yielded a total raw read count of 177,089,775. Further detail about cohort composition, data collection, and gene sequencing protocols have been previously described^37,39^. The HELIUS study was approved by the medical ethics committee of the Amsterdam University Medical Center, and all participants provided informed consent prior to enrollment in the study. Experiments were performed in accordance with all relevant regulations and guidelines, as approved by the Amsterdam University Medical Center and Declaration of Helsinki (6th, 7th revisions).

### Ethnicity

A person was defined as of non-Dutch ethnic origin if he/she fulfilled one of two criteria: (1) he/she was born outside of the Netherlands and had at least one parent born outside the Netherlands (first generation) or (2) he/she was born in the Netherlands but both parents were born outside of the Netherlands (second generation). For the Dutch samples, people who were born in the Netherlands and whose parents were born in the Netherlands were invited. The country of birth indicator for ethnicity was limited in that people who were born in the same country might be of different ethnic background, which in the Dutch context was applicable to the Surinamese population. Therefore, after data collection, participants of Surinamese ethnic origin were further classified by self-reported ethnic origin (obtained by questionnaire) as ‘African’, ‘South-Asian’, ‘Javanese’, or ‘other’.

### Microbial community composition

Paired-end reads were input into a DADA2 (v1.16)^40^ workflow to generate microbial compositions. All parameters were kept at default values except those described here. First, primers were removed from sequence reads. Then, reads were quality filtered and truncated. Two bp errors were allowed per 250 bp read, and read-ends were trimmed down to a quality score threshold of 30^41^. Then, reads were dereplicated and merged. A minimum overlap of 30 bp was required for merging to occur. After merging, chimeric sequences were removed from the amplicon sequence variants (ASVs). Lastly, taxonomy was assigned via queries to the SILVA 16S rRNA gene reference database (v138.1)^42^. Species-level phylogenetic classification was attained for 44.8% of the ASVs, genus-level for 47.8%, and family-level or higher (or unassigned) for the remaining 7.4%. A Mann–Whitney *U* test, or Wilcoxon rank-sum test, was calculated between the relative abundances (RAs) of each taxon to determine if significant differences in a particular taxon existed between the two ethnic groups.

### Principal coordinate analysis

To estimate the degree of differentiation between the common core microbiota^43^ of the Dutch and SAS samples, Principal Coordinate Analysis (PCoA)^44^ was applied using Bray–Curtis distance^45^ based on the RAs of the taxa. First, a prevalence threshold of 50% was applied within each group. Then, the first two principal components were plotted in a two-dimensional space. The PCoA was supported by a PERMANOVA analysis^46^.

### Microbiota diversity

To measure the microbial diversity within each sample, the following alpha diversity indices were computed: Chao richness^47^, Shannon^48^, Fisher^49^, and inverse Simpson^50^. Weighted UniFrac distances^51,52^ were computed to estimate beta diversity, or how different the samples within each ethnic group were from one another in terms of phylogeny and abundance.

### Biomarker analyses

To identify potential biomarkers that could distinguish the Dutch and SAS gut microbiota, a linear discriminant analysis of effect size, or LEfSe analysis^53^, was performed (*p* < 0.05 and LDA effect size > 1). LEfSe proposes microbial biomarkers based on RA, effect size, and biological consistency. It can also rank the significance of the biomarkers, i.e., taxa, because it calculates the effect size of each. This rank of significance is provided via the LDA score. LEfSe is especially useful for determining significant differences in taxa with low RAs, which is difficult to do using pure abundance data alone.

Additionally, a differential expression analysis for sequence count data, or DESeq2^54,55^, algorithm was used to calculate if significant differences existed between the bacterial abundances of the two ethnic groups. Lastly, to determine likely species- and strain-level taxonomic identities of the proposed biomarkers, the nucleotide sequences of the corresponding ASVs were input into a BLAST^56^ search for similar sequences contained only in rRNA/ITS databases.

### Network analysis

To estimate two-way ecological relationships in the Dutch and SAS microbiota^57^, we built microbial co-occurrence (social) networks^58^ using RA data at the lowest possible taxonomic classification level. We computed SparCC^59^ correlations between each pair of taxa (*p* < 0.01). Results were displayed as a network, with nodes representing taxa (size proportional to RA), and edges representing correlation (green = positive, estimating cooperation; red = negative, estimating competition). Networks were visualized using the Fruchterman-Reingold algorithm^60^ to clarify community structure.

### Ethics approval and consent to participate

The HELIUS study was approved by the medical ethics committee of the Amsterdam University Medical Centre, and all participants provided informed consent prior to enrollment in the study.

## Results

### Cohort analysis

This study analyzed a strictly age- (53 to 55 years-old) and gender-matched subset of the HELIUS gut microbiome cohort to account for any potential confounding effects of these variables on gut microbiota^61,62^. Only the Dutch and SAS ethnicities were analyzed because these were reported to have the lowest (five %) and highest (21.5%) prevalence of T2DM, respectively, among the ethnic groups of the HELIUS study^63^. After matching, 41 Dutch and 43 SAS subjects were analyzed. 34.9% of the SAS and 4.9% of the Dutch individuals included in our study were afflicted by T2DM (Table 1).

**Table 1:** Glycemic status by ethnicity.

Glycemic status	Dutch		South-Asian Surinamese	
	n	%	n	%
Diabetes	2	4.9	15	34.9
Prediabetes	15	36.6	15	34.9
Normoglycemic	24	58.5	13	34.9
Total	41	100	43	100

Subjects were classified as diabetic if their fasting plasma glucose (FPG) was ≥ 126 mg/dL or HbA1c ≥ 6.5%, prediabetic if FPG ≥ 100 mg/dL and < 126 mg/dL or HbA1c ≥ 5.7% and < 6.5%, and normoglycemic if FPG < 100 mg/dL and HbA1c < 5.7%.

### Diversity analyses

**Figure 1:**
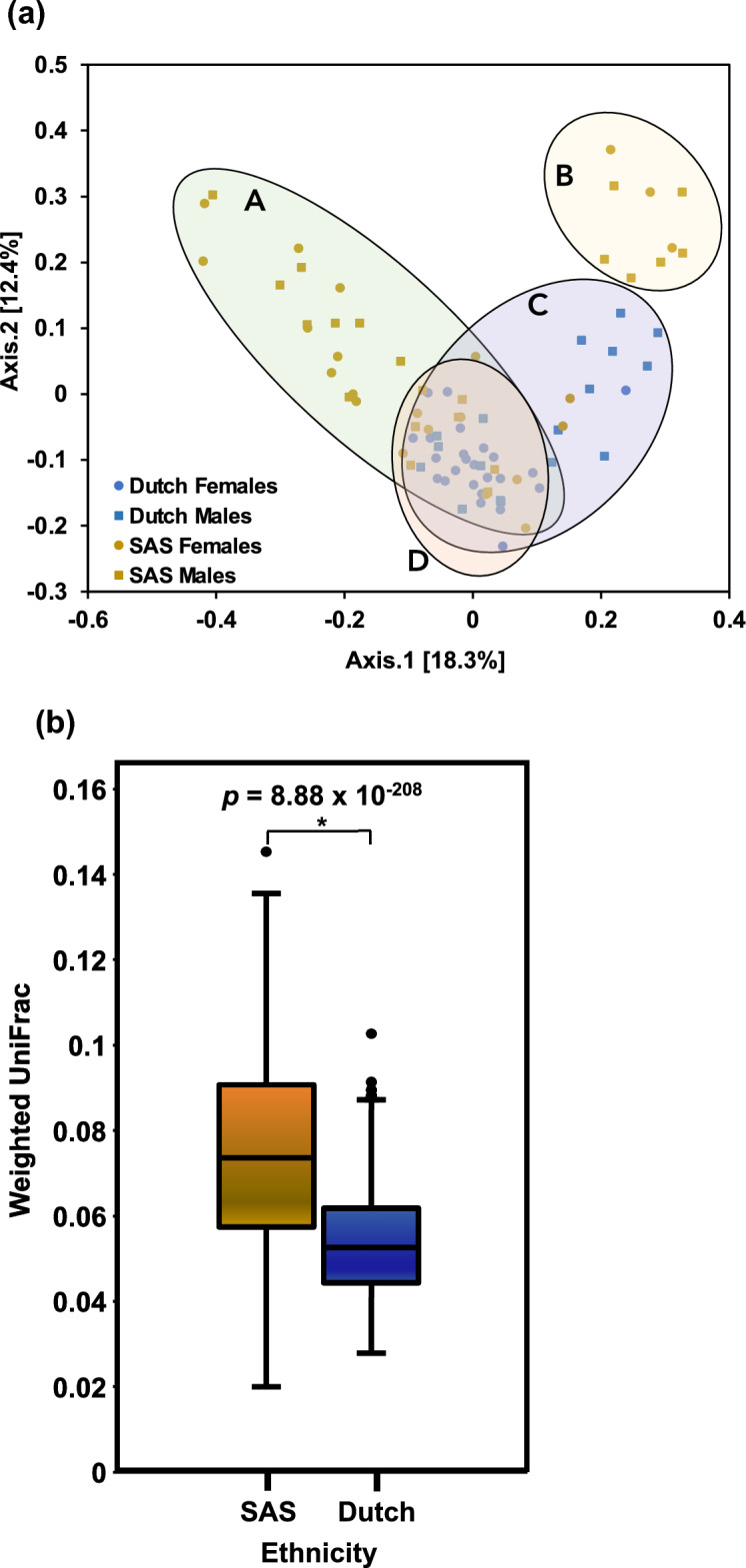
Microbial diversity between samples: (**a**) To estimate the degree of differentiation between each ethnicity’s common core microbiota, which was approximated by the top 25 most abundant taxa, PCoA^44^ was applied based on the relative abundances. First, a prevalence threshold of 50%^111^ was applied. Then, the first two principal components were plotted in a two-dimensional space. The PCoA showed that the Dutch had more similar gut microbiomes regarding the quantitative presence of the common core taxa. Contrarily, the SAS varied much more from person to person. Qualitatively, the SAS formed two clusters (A and B), and the Dutch formed a single cluster (C). Microbiota from the Dutch women formed the tightest cluster (D). There is some overlap between all the groups. (**b**) To estimate beta diversity, or how different the samples within each ethnic group were from one another for phylogeny and abundance, weighted UniFrac distances^51,52^ were computed. On average, the Dutch had a significantly more consistent gut microbiota between samples than did the SAS (*p* = 8.88 × 10^–208^).

To estimate the degree of differentiation between the common core microbiota of each ethnicity, PCoA^44^ was applied based on the RAs of the top 25 most abundant taxa (Fig. 1a). The Dutch had a more similar core gut microbiota to each other as far as abundance goes. Contrarily, the SAS varied much more from person to person. Qualitatively, the SAS abundances formed two distinct clusters while the Dutch formed a single, tight cluster. Within the Dutch cluster, the Dutch females formed an even tighter cluster. There was some overlap between both ethnicities and genders. These qualitative observations were well-supported by a PERMANOVA analysis^46^ (Table 2).

**Table 2:** PERMANOVA analysis.

Group 1	Group 2	F-score	*p*-value
SAS, female	SAS, male	0.86	0.43
SAS, female	Dutch, male	1.43	0.26
SAS, female	Dutch, female	2.83	0.07
SAS, male	Dutch, male	0.54	0.58
SAS, male	Dutch, female	0.23	0.79
Dutch, male	Dutch, female	1.48	0.22
All male	All female	2.37	0.11
All SAS	All Dutch	1.8	0.17

The PERMANOVA statistical analysis illustrates the degree of separation between individual groups. The Dutch females experienced the greatest separation from the SAS females in terms of F-score and *p*-value. This analysis also supports the higher level of compositional variability in the SAS compared to the Dutch.

Four different metrics of alpha diversity were computed (Table 3) to achieve consensus on the overall trend in microbial diversity across the samples, as each measurement of alpha diversity can be biased because this index encompasses both species richness and evenness. Chao1 estimates species richness. The Shannon and Simpson indices measure both richness and evenness, with Shannon focusing more on richness and Simpson more on evenness. Though Shannon has been shown to underestimate alpha diversity^64^. All metrics indicated that the Dutch gut harbored a significantly more diverse microbial milieu (*p* < 0.001). Weighted UniFrac distances^51,52^ between samples were computed to approximate beta diversity (Fig. 1b). A lower UniFrac distance indicates a greater degree of similarity between two sets of taxa in terms of phylogeny and abundance. The Dutch had significantly more similar gut bacterial communities to each other than did individuals of SAS origin (*p* = 8.88 × 10^–208^).

**Table 3:** Alpha diversity.

Alpha diversity metric	South-Asian Surinamese	Dutch	*p-*value
Chao1	151.51	267.8	1 × 10^–14^
Shannon	3.7	4.43	2.27 × 10^–13^
Fisher	19.39	36.29	2.57 × 10^–14^
Inverse Simpson	23.15	44	1.55 × 10^–10^

Alpha diversity is a measurement of species diversity within a sample. A two-tailed, unpaired t-test was performed between each alpha diversity metric of both ethnicities. All *p*-values < 0.005.

### Microbial abundances

**Figure 2:**
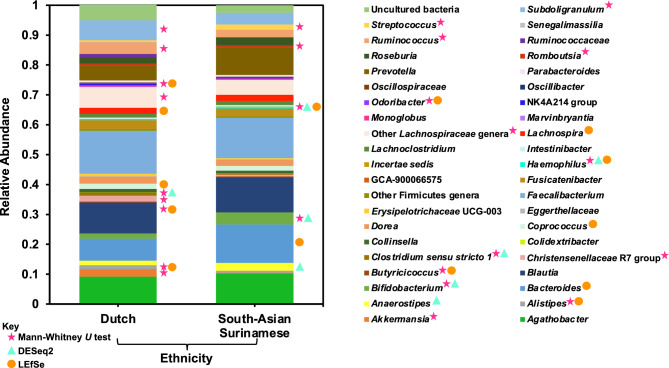
Relative abundances of common core gut microbiota: the common core gut microbiota of the Dutch and South-Asian Surinamese ethnic groups were represented by each ethnicity’s 25 most abundant genera, or otherwise lowest possible phylogenetic rank. Relative abundances were computed by DADA2 (v1.16)^40^. The blue triangles denote the taxa detected as significantly differentially abundant by a DESeq2 analysis^54,55^, the orange circles denote those identified as potential biomarkers by LEfSe, and the pink stars represent taxa found to be in significantly different abundance by a Mann–Whitney *U* test. Taxa highlighted by more than one test of differential abundance are more likely to truly differentiate the two groups.

DADA2^40^ was used to infer gut microbiome compositions. The common core gut microbiota of the Dutch and SAS were represented by the top 25 most abundant genera (Fig. 2), or otherwise lowest possible phylogenetic rank, because of the limitations of identifying finer phylogenetic resolutions with short-read, targeted 16S sequencing^43,65^. Several tests of differential abundance (DA) between the two ethnicities were performed because methods for approximating DA largely vary in output. So, it is best to perform several to attain consensus on the differentially abundant taxa, or potential biomarkers^66^.

First, a Mann–Whitney *U* test between each of the shared common core taxa was calculated (*p* < 0.05 with Benjamini–Hochberg correction). The following taxa had significantly higher average RAs in the Dutch guts: *Subdoligranulum, Ruminococcus*, a group of unclassified *Lachnospiraceae* genera, *Christensenellaceae* R7 group, *Alistipes*, *Closridium* sensu stricto* 1, Odoribacter, Butyricicoccus,* and *Akkermansia*. The following taxa had significantly higher average RAs in the SAS guts: *Streptococcus, Romboutsia, Haemophilus*, and *Bifidobacterium.*

The second test of DA performed was a DESeq2 analysis^54,55^. In the Dutch samples, *Clostridium* sensu stricto* 1* was found to be in significantly higher abundance while *Bifidobacterium, Haemophilus,* and *Anaerostipes* were found to be in significantly higher abundances in the SAS samples (*p*-values < 0.05).

**Figure 3:**
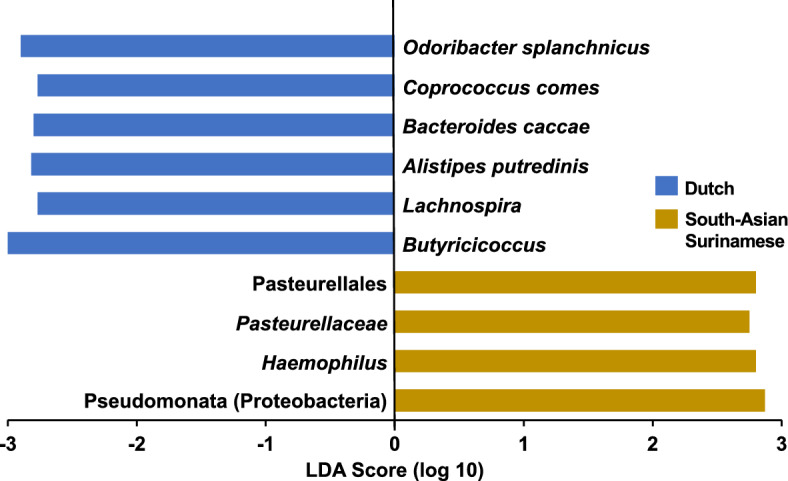
LEfSe biomarker analysis: to identify potential microbial biomarkers that could distinguish the Dutch and SAS gut microbiomes, a LEfSe analysis^53^ was performed (*p* < 0.05 and LDA effect size > 1). The LEfSe was set to propose discriminating taxa at the lowest possible phylogenetic level. The LDA score for each potential biomarker is displayed as a histogram, with all scores falling between |2.5—3|. The proposed biomarkers of the Dutch gut microbiome included *Odoribacter splanchnicus, Coprococcus comes, Bacteroides caccae, Alistipes putredinis, Lachnospira,* and *Butyricicoccus*. *Haemophilus* and its ascending phylogenetic ranks discriminated the SAS gut.

Lastly, a LEfSe^53^ biomarker analysis was computed (Fig. 3). The LDA scores assigned by the LEfSe estimate the individual contribution of each bacterium to the overall uniqueness of the community. Per the LEfSe, the discriminating bacterial biomarkers of the Dutch gut included *Odoribacter splanchnicus, Lachnospira, Bacteroides caccae, Alistipes putredinis, Coprococcus comes,* and *Butyricicoccus*. *Haemophilus* and its ascending phylogenetic ranks characterized the SAS gut.

**Figure 4:**
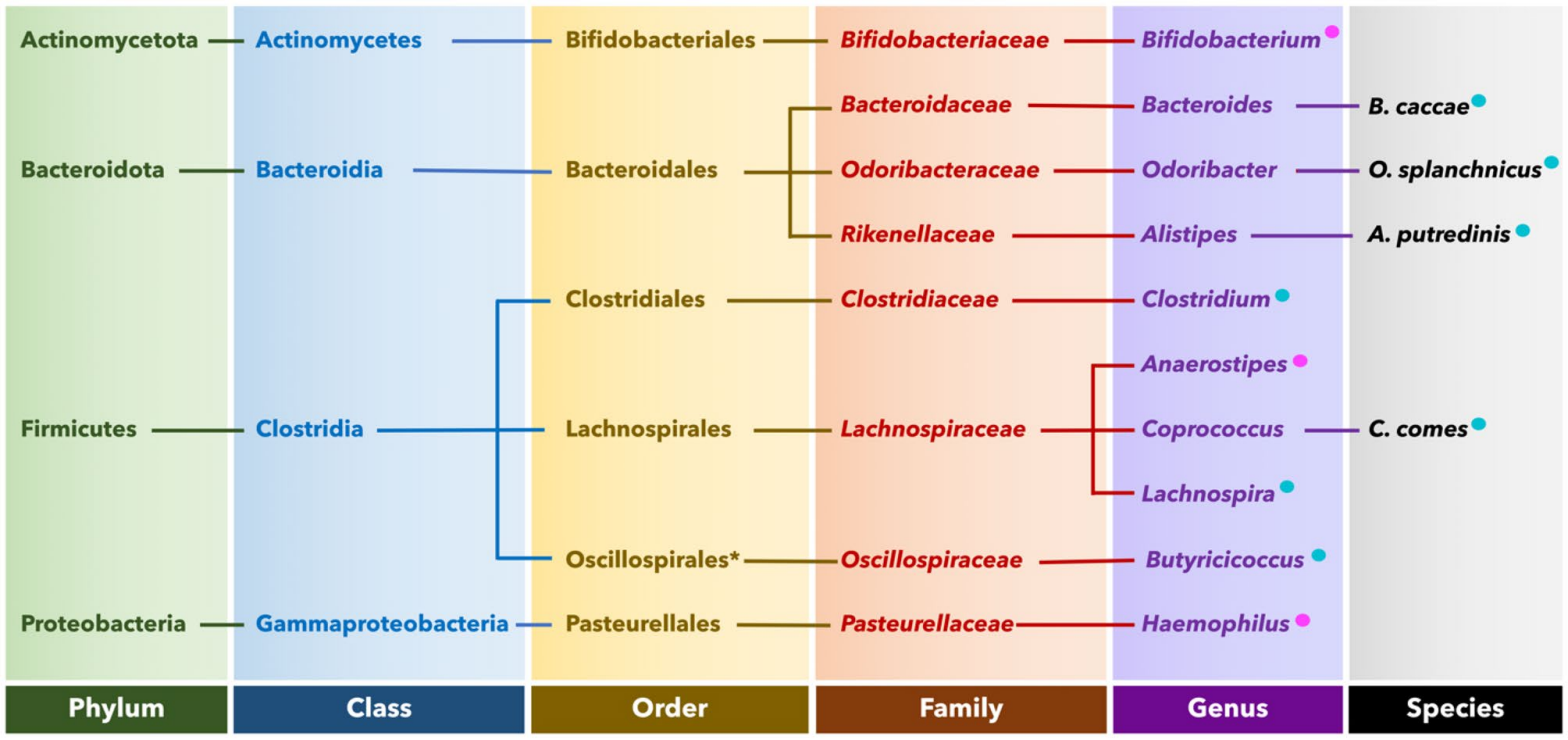
Phylogenetic tree of the proposed gut microbial biomarkers: the phylogenetic lineages of the taxa that were found to distinguish the gut microbiomes of the Dutch and SAS ethnic groups, as per the LEfSe^53^ or DESeq2^54,55^ analyses, are shown. The blue circles represent microbes that are potential biomarkers of the metabolically healthier Dutch, and the pink circles represent those of the more T2DM-afflicted SAS.

The full phylogenetic lineage of each differentiating taxon proposed by the DESeq2 and LEfSe analyses is provided (Fig. 4). We also inferred the most likely species- and strain-level identities of the differentially abundant taxa by performing a BLAST^56^ search of the nucleotide sequence of each corresponding ASV uncovered in our sequencing (Table 4). There was notably more species and strain diversity in *Clostridium* sensu stricto *1* and *Bifidobacterium* as compared to all the other discriminating taxa. Size factors for DESeq2 ranged from 0.23 to 3.08 for genus level and 0.25–3.47 for species level.

**Table 4:** Species- and strain-level identities of the differentiating taxa.

Method	Genus	Species	Strain(s)*
LEfSe taxa	*Odoribacter*	*O. splanchnicus*	DSM 201712, JCM 15291
	*Bacteroides*	*B. cacccae*	ATCC 43185, JCM 9498
	*Alistipes*	*A. putredinis*	JCM 16772
	*Lachnospira*	*L. eligens*	ATCC 27750
	*Coprococcus*	*C. comes*	FDAARGOS
	*Butyricicoccus*	*B. faecihominis*	KS-2
	*Haemophilus*	*H. parainfluenzae*	ATCC 33392
DESeq2 taxa	*Clostridium* sensu stricto *1 (Clostridium)*	*C. jeddahitimonense*	CL-2
		*C. sardiniense*	DSM 2632, JCC
	*Bifidobacterium*	*B. bidum*	NBRC 100015
		*B. colobi*	80T4
	*Haemophilus*	*H. parainfluenzae*	ATCC 33392
	*Anaerostipes*	*A. hadrus*	DSM 3319

*Some species matched with equal likelihood to multiple strains as per a BLAST search of the nucleotide sequence from the respective amplicon sequence variant.

**Figure 5:**
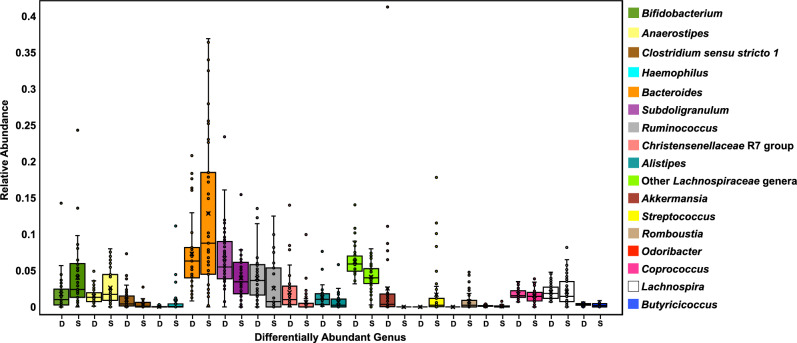
Per sample distribution of relative abundances of differentiating taxa: the per sample distributions of the RAs of the differentially abundant genera (*p*-values < 0.05), as determined by either a Mann–Whitney *U* test, DESeq2 analysis, and/or LEfSe biomarker analysis were plotted as a box and whisker plot. The Mann–Whitney *U* test highlighted *Subdoligranulum, Ruminococcus*, a group of unclassified *Lachnospiraceae* genera, *Christensenellaceae* R7 group, *Alistipes*, *Clostridium* sensu stricto *1, Odoribacter, Butyricicoccus,* and *Akkermansia* as being significantly enriched RAs in the Dutch while *Streptococcus, Romboutsia, Haemophilus*, and *Bifidobacterium* as being significantly enriched in the SAS guts. DESeq2 identified *Clostridium* sensu stricto *1* as significantly differentially abundant in the Dutch and *Bifidobacterium, Haemophilus,* and *Anaerostipes* as significantly differentially abundant in the SAS. Per the LEfSe, the biomarkers of the Dutch gut included *Odoribacter splanchnicus, Lachnospira, Bacteroides caccae, Alistipes putredinis, Coprococcus comes,* and *Butyricicoccus* while *Haemophilus* characterized the SAS gut.

The per sample distributions of the RAs of the differentially abundant taxa, as determined by either a Mann–Whitney *U* test, DESeq2 analysis, and/or LEfSe biomarker analysis were then plotted (Fig. 5). *Clostridium* sensu stricto *1* was entirely absent from the SAS guts while *Streptococcus, Akkermansia,* and *Romboustia* were totally missing from the Dutch guts. Some bacteria significantly varied in RA between individuals of one ethnicity. *Bacteroides*, one of the most abundant taxa, showed the most person-to-person variation in both ethnic groups, but this was more pronounced in the SAS. *Subdoligranulum* was widely distributed across the SAS. The RAs of *Streptococcus* in the SAS gut had the most outliers. The middle quartiles of the RAs for a group of unclassified *Lachnospiraceae* genera were almost completely non-overlapping between the two ethnic groups.

### Co-occurrence network analyses

**Figure 6:**
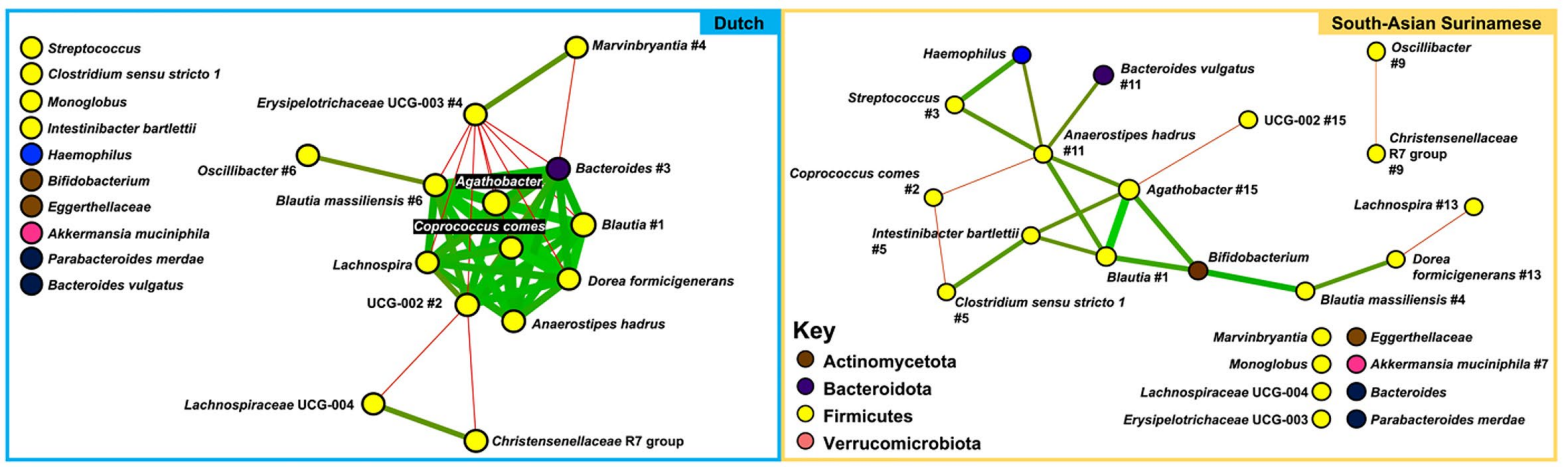
Bacterial co-occurrence networks: bacterial co-occurrence (social) networks of the Dutch and SAS gut microbiomes are shown. RA data was used at the species or next lowest possible taxonomic classification level. These networks were constructed from pairwise correlation (SparCC^59^, *p* < 0.01) matrices using Cytoscape (v3.10.1), and visualized using the Fruchterman-Reingold algorithm^60^ to clarify community structure. Each node is a distinct ASV, and the node colors represent different phyla as shown in the key. The thickness of the lines, or edges, indicates correlative strength, with green lines representing positive and red lines representing negative correlations. The numbers next to many of the organisms are centrality scores, which indicate how important that taxon is to the overall community.

To depict the ecological framework of each ethnic group’s gut microbiome, bacterial co-occurrence networks were constructed (Fig. 6), as previously described^58^. The thickness of the lines, or edges, is indicative of correlative strength. Green lines represent positive and red lines represent negative correlations. The number next to some of the organisms is a centrality score^67^, which indicates how important that taxon is to the overall community structure. At a macroscopic view, the community is tightly knit with many positive correlational relationships between taxa in the Dutch. This interconnectedness was undetectable in the SAS.

*Blautia* appears to be the most integral group for the gut of both ethnicities, since this genus was ranked as the most central taxon in both networks^68^. Subsequent Affinity Propagation^69^ analysis revealed the sixth ranked node in the Dutch network, *Blautia massiliensis*, as the centroid of this large cluster.

*Coprococcus comes* was ranked as the second most central node in SAS and was unranked in the Dutch network. In the SAS, *C. comes* negatively correlated with two other taxa (*Anaerostipes hadrus* and *Clostridium* sensu stricto *1*). In the Dutch, this bacterium positively correlated with eight other taxa (*Agathobacter, Bacteroides, Blautia massiliensis, Blautia, Anaerostipes hadrus, Dorea formicigenerans, Lachnospira,* and UCG-002) and negatively correlated with one other taxon (*Erysipelotrichaceae* UCG-003). *Oscillibacter* was another group that changed its direction of correlation with other bacteria between the two ethnicities, as it held a positive correlation with *Blautia massiliensis* in the Dutch and a negative correlation with the *Christensenellaceae* R7 group in the SAS.

*Haemophilus* and *Bifidobacterium* shared several positive edges with multiple other highly ranked nodes in the SAS network, and, in the Dutch, these genera were completely disconnected from the other nodes. In the SAS, *Haemophilus* shared positive edges with *Streptococcus* (ranked third) and *Anaerostipes hadrus* (ranked eleventh) while *Bifidobacterium* shared positive edges with *Blautia* (ranked first), *Blautia massiliensis* (ranked fourth), and *Agathobacter* (ranked 15th). A similar phenomenon occurred with *Bacteroides* and *Erysipelotrichaceae* UCG-003.

In the Dutch network, *Erysipelotrichaceae* UCG-003 (ranked fourth) was negatively correlated with eight other taxa (*Agathobacter, Bacteroides, Oscillibacter, Blautia, Dorea formicigenerans, Lachnospira*, and UCG-002), and positively correlated with one other taxon (*Marvinbryantia*). Also in the Dutch, *Bacteroides* (ranked third) held eight positive edges (*Agathobacter, Coprococcus comes*, UCG-002, *Anaerostipes hadrus, Lachnospira, Dorea formicigenerans, Blautia*, and *Blautia massiliensis)* and two negative edges (*Marvinbryantia* and *Erysipelotrichaceae* UCG-003). In the SAS, *Bacteroides* and *Erysipelotrichaceae* UCG-003 were not correlated with any other nodes.

## Discussion

The T2DM gut microbiome has been shown to be distinctly different from that of normoglycemic, insulin-sensitive individuals^14–16,21^. However, consensus on which taxa are enriched versus depleted as compared to healthy controls has yet to be reached^23^. Higher microbial production of SCFAs in the gut lumen is protective against T2DM^26,27^. The dynamics of SCFA-producing taxa have not been well-documented in minority populations because many microbiome studies are largely based on cohorts consisting of ethnic majority groups. It is important to understand how these taxa change with insulin resistance across all populations to develop effective and equitable microbiome-based therapeutics. In this study, the differential bacteria and ecological framework of the gut microbiota from two ethnic groups, the Dutch and South-Asian Surinamese (SAS), living in the same city (Amsterdam, the Netherlands) were analyzed. These two groups, both from the HELIUS cohort^37^, were compared as the SAS had a much greater prevalence of T2DM. Microbiota compositions were generated via DADA2 (v1.16)^40^. Abundances were clustered using PCoA^44^. Metrics of alpha- and beta-diversity were computed. A Mann–Whitney *U* test and two algorithms, DESeq2^54,55^ and LEfSe^53^, were used to estimate differentially abundant taxa, or potential biomarkers. Lastly, bacterial co-occurrence networks, which can extract simple patterns from complex microbial community data^58^, were constructed to compare gut microbial ecology.

### Distinct SCFA-producing gut microbial milieus between ethnicities

The DESeq2 identified several taxa that are capable of SCFA production to be significantly enriched in the SAS gut. These included *Bifidobacterium*, *Anaerostipes*, and certain strains of *Haemophilus*^70–75^. *Clostridium *sensu stricto* 1* was detected as significantly enriched in the Dutch. It is unclear if this is a SCFA-producing taxon^76–78^. Contrarily, the LEfSe (Fig. 3) discriminated the Dutch gut from that of the SAS by multiple SCFA-producers: *Butyricicoccus, Coprococcus comes, Lachnospira*, and *Odoribacter splanchnicus*^79–84^*. Alistipes putredinis* was also proposed as a biomarker of the Dutch gut, but this species produces SCFAs in small quantities^85^. Other than *Haemophilus*, of which only certain strains produce SCFAs^75^, no SCFA-producing taxa were proposed as biomarkers of the SAS gut by the LEfSe.

It is unclear from these different results if the overall abundances (Fig. 5) of SCFA-producing taxa truly vary between the two ethnicities. Though, of the potential biomarkers identified, *Haemophilus parainfluenzae* ATCC 33392 (Table 4) would likely serve as the best discriminating bacterium between these two ethnicities. A consensus approach using various DA methods has been proposed as the best way to identify truly differential taxa. Compared to other DA tests, LEfSe has been shown to have a higher false discovery rate and predisposition to identify more abundant taxa as differential^66,86^. It has also been demonstrated that the Mann–Whitney *U* test has a comparatively higher false positive rate^66^. Contrarily, DESeq2 has a lower type I error rate while maintaining moderate sensitivity^87,88^.

Given these biases, *Haemophilus* is likely the most robust biomarker because of the concordance among our DA tests on its significance and it being of relatively low RA in both groups. *Odoribacter* is also likely a robust biomarker given its low RA across both ethnicities. *Bacteroides* may be a false call by LEfSe because it was one of the most abundant taxa in both ethnicities. Finally, *Akkermansia*, enriched in the Dutch, and *Streptococcus,* enriched in the SAS, might be notable biomarkers even though they were only called by the Mann–Whitney *U* test. This is because of their complete absence across most of the subjects of each respective ethnicity, as LEfSe and DESeq2 are unable to model zeros^88^. In future work, it would be beneficial to add more specific methods, such as ALDEx2^89^ or ANCOM-BC^90^, to identify differential taxa with greater certainty. While SCFA-producers may not be present in significantly different quantities between the Dutch and SAS, the Dutch gut contained a more phylogenetically diverse SCFA-producing microbial milieu.

*Haemophilus parainfluenzae*, a known respiratory pathobiont, is also a gut pathobiont, as it plays a pro-inflammatory role in Crohn’s Disease^91,92^. A higher abundance of *Haemophilus parainfluenzae* has been positively correlated with obesity and cardiometabolic disease by some^93^, but negatively associated with similar entities by others^94,95^. To the best of our knowledge, *Haemophilus parainfluenzae* has not been clearly shown to produce SCFAs in a significant quantity. Our work indicates that this bacterium could be significantly associated, or even a pathobiont, in the guts of those with risk factors for metabolic disease, especially in ethnic minorities. Overall, our results indirectly indicate that normo- and hyperglycemic patients are unable to be distinguished based on microbial SCFA production.

### A more complex gut microbiome ecological framework in the ethnic majority

Our PCoA (Fig. 1a) displayed that RAs and phylogeny were more similar across the Dutch samples as compared to the SAS. Sex was not observed to significantly impact gut microbiota because each PCoA cluster contained a roughly equal proportion of both sexes. This is consistent with previous work that has failed to demonstrate a clear relationship between sex and the gut microbiome^62^. Additionally, the overlap between the Dutch and SAS abundances may be explained by how long ago the SAS individuals had immigrated to the Netherlands, as over time they likely increasingly incorporated Dutch foods and were exposed to the Dutch environmental microbiome.

Bacterial co-occurrence, or social, networks of the Dutch and SAS gut microbiota were computed (Fig. 6). Network analyses can help determine the ecological core microbiota, i.e., which taxa are key for community structure and, possibly, function^96^. Compared to the SAS, the intra-taxonomic relationships in the Dutch gut were more interconnected, with many more positive correlational relationships. The Dutch network also contained a large cluster of SCFA-producing taxa (*Coprococcus comes, Lachnospira, Blautia,* and *Haemophilus*). This positively interconnected ecological framework correlates with the significantly higher average alpha diversity (Table 3), lower beta diversity, and more stable community composition and phylogenetic distribution across the Dutch samples (Fig. 1). Previous studies have associated the SAS and diabetics with a less diverse gut microbiota^23,97^.

Our networks support the notion that the role played (cooperation versus competition) by a taxon may change with ethnicity and is possibly a function of host glycemic phenotype. In the Dutch network, *Erysipelotrichaceae* UCG-003 was estimated to be the fourth most important taxon for the community structure and had several negative relationships with other taxa. However, in the SAS network, this taxon was unranked and lacked any correlations with other community members. *Erysieplotrichaceae* UCG-003 has previously been positively associated with insulin resistance and obesity^98,99^. *Erysieplotrichaceae* UCG-003 has also been proposed as a marker of healthy aging^100^. This taxon may be negatively correlated with others in the Dutch network because there is competition for a mutual resource. In the Dutch, the other likely beneficial taxa that *Erysieplotrichaceae* UCG-003 is correlated with may be better able to acquire that resource and use it to produce a metabolite that is beneficial for the host, such as a SCFA. This competition was not observed in the SAS.

The Dutch, less afflicted by T2DM, may have a more interconnected gut microbial ecology because of greater bacterial cross-feeding interactions. Cross-feeding is dependent on microbiota spatial organization and is critical for SCFA production^26,27^. Several taxa that were either SCFA producers or negatively associated with T2DM were members of the large, interconnected Dutch network. *Bacteroides,* which has a strong negative association with T2DM^22,24,101^, was disconnected and unranked in the SAS network, but was ranked as the third most integral taxon in the Dutch network. So, the metabolically beneficial role of this bacterium is likely a reproducible and accurate finding. *Oscillibacter,* ranked sixth in the Dutch and ninth in the SAS network, has been shown to be positively associated with T2DM^102,103^. *Bifidobacterium*, which has been reported to be protective against T2DM^13,22^, had more positive relationships with other bacteria in the SAS group, but was unranked in both networks. So, the role of a particular taxon in relation to metabolic fitness may depend on host ethnicity.

The main limitation of our study was that we were unable to stratify per-sample the two ethnicities by diabetic status. Although subjects were strictly age- and gender-matched, other factors, such as ethnically variable genetic predispositions^104,105^, epigenetics^106,107^, diet^61,108,109^, and socioeconomic status^110^ likely confounded our findings. The ethnic minorities of the HELIUS cohort were of lower socioeconomic status, which significantly impacts health equity^39^. Differences in dietary and exercise patterns between the ethnicities were also observed in the HELIUS study^39^. Additionally, most of the SAS were first-generation immigrants, so they had a shorter length of residence in the Netherlands^37^. More work is needed to understand gut microbiota in the context of each ethnicity to drive forward personalized medicine for metabolic diseases.

## Data Availability

The HELIUS data are owned by the Amsterdam University Medical Centers, location AMC, in Amsterdam, the Netherlands. Any researcher can request the data by submitting a proposal to the HELIUS Executive Board, as outlined at http://www.heliusstudy.nl/en/researchers/collaboration, by email to heliuscoordinator@amsterdamumc.nl. The HELIUS Executive Board will check proposals for compatibility with the general objectives, ethical approvals, and informed consent forms of the HELIUS study. There are no other restrictions to obtaining the data and all data requests will be processed in the same manner. The microbial genomic sequences from the HELIUS cohort, which were used for this study, are stored under protected access on the European Genome-Phenome Archive (https://ega-archive.org/datasets/EGAD00001004106).

## Abbreviations

ASV: Amplicon sequence variant
BP: Base-pair
DA: Differential abundance
DESeq: Differential expression analysis for sequence count data
FPG: Fasting plasma glucose
GLP-1: Glucagon-like peptide 1
HELIUS: Healthy life in an urban setting
PCoA: Principal coordinate analysis
RA: Relative abundance
SAS: South-Asian Surinamese
SCFA: Short-chain fatty acid
T2DM: Type two diabetes mellitus

## Code availability

Our entire downstream analysis through the open-source PluMA^11^ initiative is available at http://biorg.cs.fiu.edu/pluma/pipelines.html.
